# Long-term athlete development in schools: a systematic review and narrative synthesis

**DOI:** 10.3389/fspor.2025.1706060

**Published:** 2025-11-20

**Authors:** Nele Söker, Jasper Schamerowski, Astrid Zech, Simon Steib, Lina Rahlf

**Affiliations:** 1 Department of Human Movement and Exercise Physiology, Institute of Sport Science, Europa-Universität Flensburg, Flensburg, Germany; 2 Department of Human Movement and Training Science, Institute of Psychology and Human Movement Science, Hamburg University, Hamburg, Germany; 3 Department of Human Movement, Training and Active Aging, Institute of Sports and Sports Sciences, Heidelberg University, Heidelberg, Germany

**Keywords:** sport school, youth sport, talent programmes, education, gifted

## Abstract

**Introduction:**

Evidence systematically assessing the effects of sports schools on long-term athlete development is largely lacking. In general, sport schools play an increasingly important role in the development of new Olympic and top athletes. Additionally, talent programmes for the context of schools are a strategy for identifying and developing talent in sport. Both approaches are designed to promote long-term development of talent in sport, but benefits remain unclear. This study examines the development of athletes in the school environment within the framework of Long-Term Athlete Development (LTAD).

**Methods:**

A systematic search in PubMed, Web of Science, SportDiscus was conducted following the PRISMA guidelines, incorporating the PICO strategy. Eligible trials were selected by (i) studies conducted in school (ii) including pupils (iii) conducting a quantitative design (iv) providing physiological, physical, psychological and social data, as well as sporting and academic results. Data extraction and risk of bias assessment were performed independently by two reviewers. Findings were synthesized narratively.

**Results:**

Based on 21 studies, talent-development approaches in schools positively impact physiological and physical aspects. In four studies, authors noted increased psychological stress, with female student-athletes more frequently affected. Two studies found no negative impact on short-term academic success, whereas two reported lower long-term educational outcomes for sport profile pupils.

No consistent link was found between talent programmes and long-term sporting success. Although athletes reported enhanced social support and stronger relationships with coaches and peers, the competing demands of academics and sport frequently caused social isolation beyond the sports school context.

**Conclusion:**

This narrative synthesis provides a comprehensive understanding of school-based talent development approaches on LTAD. Talent development programmes in schools support physical performance, social networks, and short-term academic success, but pose challenges for psychological aspects and long-term academic and sporting success. Better alignment with long-term athlete development aspects, including mental health support, is needed. Due to the heterogeneous methodologies applied, a systematic approach for pooled data synthesis was not feasible. Future research should address long-term outcomes and close methodological gaps.

## 1 Introduction

The development of athletic talent is a multifaceted process influenced by numerous factors. The main factors discussed in the literature focus on biological and genetic predispositions, deliberate practice and play environmental prerequisites, psychological and physical factors (1–5). In order to develop these main factors, athletes need a proper learning environment to maximize their potential (6). In the past, it was predominantly seen as the task of sports clubs and sports associations. But as the development of elite athletes is increasingly characterized by institutionalization and government involvement in order to achieve international success (7) many countries responded by implementing talent identification and development in the school system.

This research aims to globally review how schools in various countries implement talent development programs, as well as the alignment of these programs with the Long-Term Athlete Development (LTAD) framework. The divergence between countries can be attributed to fundamentally different systemic approaches: e.g., in countries such as Germany or China, talent programmes are part of state-supported elite sport schools, while in nations like the USA or Australia, athletic development is more broadly integrated into regular school or college structures, where sports are a standard component of education (8, 9). However, the heterogeneity between countries also persists in the approach within countries. E.g., some systems emphasize multi-sport environments, others focus on sport-specific academies, such as elite football schools in Germany (9). Consequently, it becomes evident that not only international but also national approaches to talent development within educational contexts span a continuum from systematically organised physical education curricula to institutionally specialised sports academies.

Several systematic reviews indicate that the objectives of school-based talent development approaches largely align with the above mentioned factors discussed in the literature on talent development, but in addition to the development of athletes potential, they also seek to ensure academic achievement and psychosocial development (10–13). Given these diverse responsibilities, it can be speculated that schools, unlike sports clubs, face more complex challenges. This suggests that many schools are not fully equipped to address these demands and therefore rely on individualised strategies for talent development. This lack of standardization was highlighted in the systematic review of Prieto-Ayuso and colleagues (11), which demonstrated missing consensus on state measures or guidelines for talent development in schools.

This finding appears particularly surprising given that effective, structured approaches to talent development already exist in the sport science literature, which schools could potentially adopt. The most well-known of these models is the Long-Term Athlete Development (LTAD) framework. The LTAD framework is designed to guide the development of athletes from early participation in sports to lifelong involvement. This framework emphasizes the progression of talent-specific key aspects, including physiological adaptions associated with growth and maturation as well as the development of psychological, and social skills (14, 15). In addition to the numerous approaches to LTAD, there is general agreement on the essential components of an LTAD programme, which include age-appropriate training, long-term monitoring of physiological, physical and psychological development, and a holistic approach to developing athletes across various domains such as strength, endurance, motor skills, and psychological resilience, as well as social and mental growth and fostering long-term performance (14). However, it remains largely unknown to what extent schools implement such standardised frameworks like the LTAD in their talent development programmes.

Despite the apparent suitability of the LTAD framework for school contexts, research suggests a concerning disconnect between theory and practice. An examination of existing development programmes in school shows that the focus is not necessarily on physical development, but on promoting and developing talent in sport, including psychological and social aspects (15) while also aiming to reduce the double burden of general school education and the narrow sport-specific training schedule (16). In theory, these aspects of the LTAD framework align with school-based approaches to talent development, as talent development in sport cannot be realized without adequate Long-Term Athlete Development.

However, there is often a gap between the implementation of talent development in schools and their alignment with evidence-based LTAD aspects (11). Enhancing physical performance in youth is a complex and dynamic issue, due to the varying interactions of growth, maturation, mental development and training and seems difficult for schools to implement, taking all aspects into account. Yet, childhood provides a unique opportunity to take advantage of a highly plastic neuromuscular system and help shape the fundamental skills, attitudes and opportunities that contribute to LTAD (17).

Understanding the outcomes of school-based talent development approaches is therefore fundamental, as early interventions and support systems can have profound implications for athletic performance, injury prevention, and lifelong participation in sports. This is in particular important because school-based talent development programmes are associated with some critical challenges. Potential negative consequences such as early specialization, overtraining, and increased psychological stress have been highlighted in the literature, which may compromise athlete's long-term development and well-being (18). These risks underscore the need for careful alignment of school programmes with evidence-based frameworks like LTAD to ensure balanced and sustainable athlete development.

Given these considerations, this systematic review seeks to evaluate the outcome of school-based talent development programmes, and their alignment with the aspects on the LTAD framework. It further examines the variables used to assess programme effectiveness. By synthesizing existing research, this study aims to provide insights into how schools can optimally contribute to fostering athletic excellence and sustainable sports participation.

## 2 Conceptual framework and talent-specific key aspects of LTAD

This systematic review and narrative synthesis is guided by talent-specific key aspects of the LTAD model. These aspects were selected to provide a structured conceptual foundation for understanding the multifaceted nature of talent development in school-based sport programs. By including both sport and academic outcomes, this framework sets the stage for the subsequent systematic analysis of dual-career development.

The talent-specific key aspects of LTAD include physiological, physical, psychological, identity and social factors, as well as sporting success (14, 17). In addition to the talent-specific key aspects, academic success is also included, given the dual demands placed on student-athletes in the school context. The key aspects are outlined and defined below to guide the synthesis of findings.

Physiological aspects refer to adaptations related to growth, maturation, and biological processes that influence an athlete's physical capabilities. These aspects focus on the physiological changes such as metabolic and cardiovascular parameters [e.g., hemoglobin levels, resting heart rate, and maximal oxygen consumption (VO₂max)] that occur as a result of training and natural development.

Physical aspects encompass the enhancement of motor skills, strength, endurance, speed, and coordination necessary for athletic performance. Psychological aspects involve the cultivation of mental resilience, motivation, self-confidence, focus, and stress management skills.

Identity and social aspects refer to the formation of an athletic self-concept and the integration of sport into an individual's personal and social life. These aspects also include relationships with coaches, peers, family, and support systems, all of which influence the athlete's development and career progression.

Additionally, long-term performance was considered, encompassing sporting success, which refers to achievements in national and international competitions. Although academic success is not a talent-specific key aspect of the LTAD approach, it is included here to reflect the dual demands of student-athletes and to provide a holistic framework for the subsequent analysis. Academic success is defined by good academic grades, cognitive test scores, and educational and career outcomes, including higher educational qualifications and university enrollment.

## 3 Methods

### 3.1 Search strategy

Two independent researchers (NSo and JS) conducted a systematic literature search including articles from January 2000 until April 2025. This time frame was selected to capture a sufficiently broad and contemporary body of research, as school systems and talent development programmes are continuously evolving.

The systematic computerized search was conducted in three databases (PubMed, Web of Science, SportDiscus). Studies in English or German, published in peer-reviewed journals and conducting a quantitative study design were taken into account. Further, studies addressing talent development in schools, including pupils and providing physiological, physical, along with anthropometric measurements, psychological and social data, as well as sporting and academic results, were included.

The search terms were defined based on the PICOS system (Patient/Problem, Intervention, Control/Comparison, Outcome, Study design) (19).

In addition to the electronic search, further appropriate studies were identified by screening the reference list of the included studies and through additional sources uncovered using a co-citation method using the bibliographic coupling concept (https://www.connectedpapers.com). The review process adhered to the Preferred Reporting Items for Systematic Reviews and Meta-Analyses (PRISMA) guidelines to ensure methodological transparency and reporting quality (19).

A detailed overview of the search process can be found in the supplementary materials.

### 3.2 Selection criteria

The selection criteria were based on the PICOS system and had to be fulfilled in the studies for inclusion to the systematic review: i) the study population had to be pupils at a sport school, regular school (RS) including sport profile or additional sport programme (P); ii) the intervention was defined as any kind of sports talent development intervention at school, such as specialized training programmes or extracurricular sport activities (I); iii) individuals or classes from RS and with regular physical education (C); iv) relevant outcomes addressed talent identification and multidimensional talent development aspects, physiological, physical, psychological, social, sporting, and academic success, following the LTAD framework (O); v) the settings were defined as sports schools, RS including sport profile or additional sport programme (S).

No restrictions were placed on age or educational level, as studies often included mixed school levels or used retrospective designs, in which former pupils were assessed after graduation. This approach was chosen to capture the full range of school-based talent development pathways.

Studies were excluded if they did not explicitly follow a talent development approach in schools, lacked a quantitative study design, were conducted in an incorrect setting (e.g., preschool, university, or club), the full-text was not available, or if they did not provide relevant data for the purpose of the study. All results were exported to rayyan.ai for the selection process (20).

After duplicates were automatically removed by rayyan.ai, two independent researchers of the team (NSo and JS) manually screened the results of the databases. This process was carried out in two phases. First, all the studies were initially included or excluded based on the content of the titles and abstracts. Second, the full-texts were read and included or excluded with regard to the criteria. The two reviewers worked independently and blinded to each other's decisions within rayyan.ai. Conflicts were automatically flagged by the software and then discussed in consensus meetings.

In case of disagreement, a third researcher of the team (LR) was added to the discussion to make a final decision. The software tool rayyan.ai assisted only in ensuring a systematic approach during the selection process, while all decisions were manually made by the researchers.

### 3.3 Risk of bias assessment

To analyze the methodological quality the Mixed Methods Appraisal Tool (MMAT), an Excel-based assessment tool, was used (21). The MMAT is a tool for the critical appraisal of systematic reviews including single or combined study designs, i.e., studies that use qualitative, quantitative or mixed methods, and was chosen over other risk-of-bias tools because it enables a consistent and comprehensive appraisal across diverse methodological approaches within a single framework. It allows for the assessment of methodological quality across five categories of studies to be assessed: qualitative research, randomised controlled trials, non-randomised studies, quantitative descriptive studies and mixed methods studies (21). It was selected as the most suitable tool, since the included studies showed a variation in study designs, including both purely quantitative and mixed methods approaches. Since quantitative studies were included, as well as the quantitative components of mixed methods studies, an appraisal tool was needed that could accommodate both types.

The validity and reliability of the MMAT has been previously reported as ranging from moderate to excellent for its specific criteria and substantial for the overall quality scores of appraised studies (22). The tool includes two initial screening questions and five methodological quality questions tailored to the specific design of the studies. Responses to each question were critically evaluated by two researchers (NSo and LR) and recorded as “yes”, “no” or “can't tell”. Disagreements were resolved through discussion. Although study quality was appraised for the entire study design, including qualitative components where present, only the quantitative data were considered in the synthesis and interpretation of results. In line with MMAT guidance each “yes” answer was scored as 1 and “no” or “can't tell” as 0. The total number of “yes” responses per study was converted into a percentage score to facilitate comparison (e.g., 100%, 80%, 60%, 40%, or 20% of quality criteria met) (22). These percentage scores were summarized descriptively rather than used for weighting in the synthesis. However, they informed the interpretation of findings by highlighting studies with stronger methodological rigor.

### 3.4 Data extraction

After using rayyan.ai for the selection process, the data collection was carried out by two authors (NSo and JS) using a custom-designed Microsoft Excel spreadsheet. Both researchers independently extracted data and cross-checked all entries to ensure accuracy and consistency. General information (such as author, country, sample size, and age), approach, study design, control group, training load, training period, sports and measurement outcomes were extracted. The approach variable reflected the contextual setting of the study and included sports schools, elite sport schools, regular schools with a sport profile, and regular schools offering an additional sport programme. Measurement outcomes covered physiological, physical, psychological, social, sporting. and academic success aspects.

### 3.5 Narrative synthesis

This review follows a systematic methodology for literature search, study selection, and quality assessment. Due to the considerable methodological heterogeneity of the included studies, including differences in study design, participant characteristics, measured outcomes, and interventions, a meta-analysis was not conducted. Instead, the findings were synthesized narratively as part of the systematic review process, following established guidelines for narrative synthesis in systematic reviews (23). The narrative synthesis involved summarizing study characteristics in a table and structuring the results according to the LTAD framework, including the identification of consistencies and contradictions as well as the exploration of relationships between key aspects. Study findings were categorized to the LTAD aspects using deductive definitions from the literature (14, 15, 17) and refined inductively based on the study data. To ensure objectivity, two reviewers (NSo and LR) worked independently and discussed findings until consensus was reached.

This narrative synthesis allowed for a more nuanced understanding of the studies, highlighting the strengths, limitations, and implications of the findings, and offering a clearer picture of the current state of knowledge on the topic. The results were structured and presented along talent-specific key aspects of talent development, as identified through the LTAD framework and derived inductively from the findings of the included studies. Through this approach, the review provides an in-depth exploration of the evidence, despite the lack of statistical aggregation through a meta-analysis.

## 4 Results

### 4.1 Trial flow

**Figure 1:**
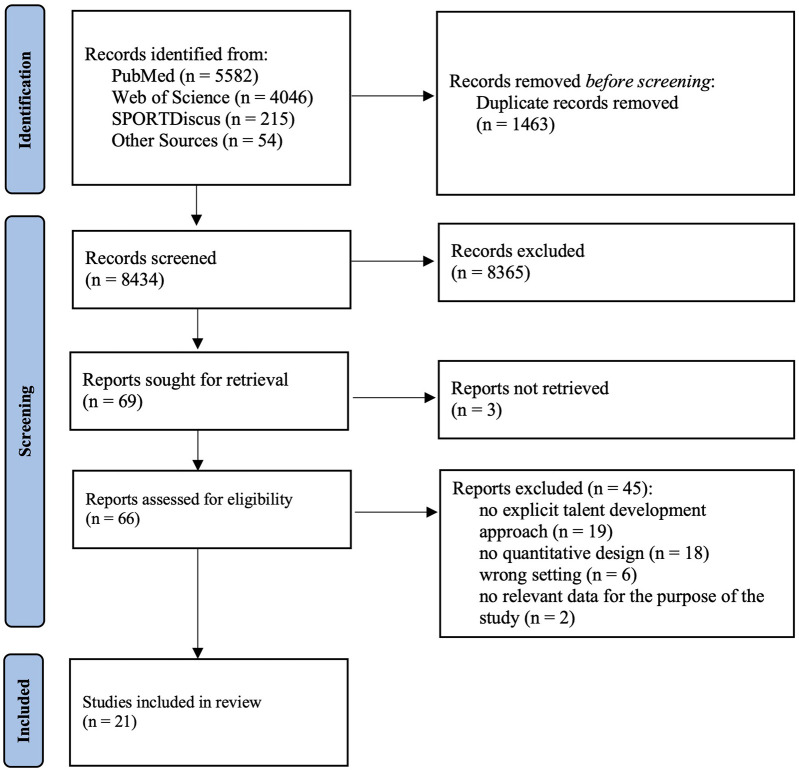
PRISMA flow diagram. Flow diagram showing the selection process of studies included in the systematic review. Numbers indicate the records identified, screened, excluded, and included at each stage.

On 16th April 2025 the final search phase was completed. 8,434 studies remained after removing duplicates. After screening the title and abstract, 8,365 were excluded and 69 were remained. Following this, the full text of the selected studies was screened using the inclusion/exclusion criteria. During this process 48 papers were excluded due to no explicit talent development approach in school (*n* = 19), no quantitative design (*n* = 18), wrong setting (preschool, university, club) (*n* = 6), full-text were not available (*n* = 3) and no relevant data for the purpose of the study (*n* = 2). After all, a total of 21 studies were able to meet the inclusion criteria. Figure 1 presents the process of study identification, screening and selection (Table 1).

### 4.2 Overview of study characteristics

Table 1 shows the characteristics of the 21 studies.

**Table 1:** Characteristics of included studies.

Study	Design	Country	Sports	Sample size (n)	Age (years), mean ± SD	Control group	Approach	Training^a^ load (hours per week)	Training period (weeks)	Measurement (talent-specific key aspects)
Brand and Hoyer (27)	cross-sectional	Germany	16 different sports	786	12–15 (range)	yes	elite sports school	20	not applicable (cross-sectional)	psychological
Davis et al. (28)	mixed methods	Sweden	alpine ski	173	17.5 ± 1.15	no	elite sports school	13.42	no data	psychological
Earle et al. (26)	case design	UK	8 different sports	75	16 ± 1.6	no	sport school	no data	no data	identity/social, psychological academic success
Emrich et al. (29)	retrospective	Germany	different olympic sports	199	no data	yes	elite sport school	no data	not applicable (retrospective)	sporting success, academic success
Granacher and Borde (30)	experimental controlled	Germany	6 different sports	20	9.5 ± 0.5	yes	elite sport school (primary school)	4.5 (only physical education)	52	physical, psychological, academic success
Güllich (31)	retrospective	Germany	athletics	213	23.4 ± 3.5/17.9 ± 1.1	yes	elite sport school	exactly stated in study^b^	not applicable (retrospective)	sporting success
Hes and Asienkiewicz (42)	longitudinal	Poland	gymnastic	253	7–9 (range)	yes	sports acrobatics classes (RS including sport profile)	12	104	physical
Kristiansen and Stensrud (24)	mixed methods	Norway	handball (female)	10	13	no	elite sports school	12–16	313	physiological
Kristiansen and Stensrud (32)	mixed methods	Norway	handball (female)	6	13	no	elite sports school	8–14	52	physiological
Nyberg et al. (44)	retrospective	Sweden	41 different sports	274	39.92 ± 6.25	yes	dual career support programme (RS including additional sport programme)	no data	not applicable (retrospective)	sporting success
Peterson et al. (33)	longitudinal	Sweden	primarily football and 11 different sports	156	13	no	sport school	no data	308	identity/social, psychological
Roth et al. (25)	repeated cross-sectional	Germany	17 different sports	11,451	10 and 13	no	sport school	no data	exactly stated in study	physical
Roth et al. (34)	longitudinal	Germany	15 different sports	1,590	10–13 (range)	no	sport school	4.84 boys 4.98 girls	156	physical
Schmid et al. (35)	retrospective	Norway	cross-country skiing	116	22–26 (range)	no	elite sport school (programme for cross-country skiing)	9.44 first two yrs. 10.42 last two yrs.	not applicable (retrospective)	psychological
Skrubbeltrang et al. (43)	longitudinal	Denmark	8 different sports	1,010	12–16 (range)	no	sport classes (RS including sport profile)	no data	104	identity/social,
Stambulava et al. (15)	mixed methods	Sweden	27 different sports	250	16	no	elite sport school	no data	30	identity/social, psychological
Stornæs et al. (37)	prospective person-centered analytical	Norway	27 different sports	946	13–14 (range)	yes	sport school and one ballet class (RS including sport profile)	no data	104	psychological
Thompson et al. (38)	mixed methods	UK	7 different sports	72	17.29 ± 0.52	no	sport school	no data	33	physical, identity/social, psychological, academic success
Thompson et al. (39)	longitudinal mixed methods case design	UK	8 different sports	83	17.40 ± 0.53	no	sport school	10.5	no data	physical, psychological
Van Rens et al. (40)	retrospective	Nethderlands	10 different sports	242	21 ± 2.8	yes	elite sport school	16	not applicable (retrospective)	sporting success, psychological, academic success
Zhao et al. (41)	mixed cross-sectional and longitudinal	China	swimming and racket sports (male)	21	12.14 ± 0.62	no	elite sport school	20.8	96	physiological, physical

a Training includes both the regular physical education classes and the additional training provided within the talent development programme in school.

b Due to the detailed presentation in the study, an overview in the table is not possible.

The research involved 21 studies conducted in Germany (*n* = 6), Norway (*n* = 4), Sweden (*n* = 4), UK (*n* = 3), China (*n* = 1), Denmark (*n* = 1), Netherlands (*n* = 1) and Poland (*n* = 1). The baseline sample sizes varied between 6 (24) and 11,451 (25), participants, resulting in a total of 17,881 subjects. Most studies were conducted in European countries, particularly in Germany and Scandinavia, reflecting the geographical focus of available research.

Eighteen studies examined sports schools (24–41), while two studies explored RS including sport profile (42, 43) and one study focused on RS including additional sport programme (44). A differentiation was made between „sports schools“ and „elite sports schools“ (ESC). The term „elite sports schools” refers to all institutions with a focus on youth performance development, including those officially named, for example, *Sports Academy* or *Top Sports School*. Overall, the majority of studies focused on sports school settings rather than alternative school-based talent models.

Two studies focused on primary school-aged children (7–10 years) (30, 42), while seven targeted middle school-aged children (11–15 years) (24, 25, 27, 34, 37, 41, 43), four on high school-aged teenagers (16–18 years) (28, 36, 38, 39), and three on both middle and high school-aged teenagers (26, 32, 33). Studies tend to focus on adolescents rather than younger age groups.

In five studies (29, 31, 35, 40, 44), no information regarding the age of the participants was provided due to the retrospective design. Five studies employed a retrospective design (29, 31, 35, 40, 44), five studies used a mixed methods design (24, 28, 32, 36, 38), and four studies utilized a longitudinal design (33, 34, 42, 43) with a training period of at least one academic year. Additionally, there was one study each using a cross-sectional design (27), experimental controlled design (30), prospective person-centered longitudinal design (37), longitudinal mixed methods case design (39), case design (26) repeated cross-sectional design (25) and a mixed cross-sectional and longitudinal design (41). Eight studies were conducted with a control group (27, 29–31, 37, 40, 42, 44). Taken together, most studies applied retrospective or mixed-methods designs, with comparatively fewer longitudinal or experimental approaches.

Twelve studies provided background information regarding training load. The volume ranged from 4.5 h (physical education only) (30) to 20.8 h (total training load) (41) per week. The following sports were examined: cross-country skiing (35), alpine skiing (28), athletics (31), gymnastics (42), two times women's handball (24, 32), swimming and racket sport (41) and fourteen times more than six sports in one study (25–27, 29, 30, 33, 34, 36–40, 43, 44). This distribution illustrates a predominance of multisport contexts among the included studies.

A total of six different talent-specific key aspects of LTAD were measured. Three studies assessed physiological aspects (24, 32, 41), seven examined physical aspects (25, 30, 34, 38, 39, 41, 42), five focused on psychological aspects (26, 27, 37, 39, 40), four investigated identity and social aspects (26, 35, 36, 38), five analyzed sporting success (29, 31, 33, 40, 44) and four assessed academic success (30, 31, 38, 40). Overall, physical aspects were investigated most frequently.

In summary, the reviewed studies predominantly focused on sports school settings and mostly employed retrospective or mixed-methods designs. Research addressing psychological, social, and academic aspects was comparatively limited, highlighting gaps in the current evidence base. This diversity in study designs allows for a comprehensive understanding of school-based talent development approaches on LTAD, although it also limits the generalizability of the findings.

### 4.3 Risk of bias assessment

The study methodological quality of the included studies is summarized in Table 2.

**Table 2:** Quality score.

Study	Design	Methodological quality criteria					Total quality assessment
		1	2	3	4	5	
Brand and Hoyer (27)	Quantitative non-randomised	Yes	Yes	Yes	Can't tell	Yes	80%
Davis et al. (28)	Quantitative descriptive	Yes	Can't tell	Yes	Can't tell	Yes	60%
Earle et al. (26)	Quantitative descriptive	Yes	Can't tell	Yes	Yes	Yes	80%
Emrich et al. (29)	Quantitative descriptive	Yes	Can't tell	Can't tell	No	Can't tell	20%
Granacher and Borde (30)	Quantitative non-randomised	Yes	Yes	Yes	No	Yes	80%
Güllich (31)	Quantitative non-randomised	Yes	No	Yes	No	Yes	60%
Hes and Asienkiewicz (42)	Quantitative non-randomised	Yes	Yes	Yes	No	Yes	80%
Kristiansen and Stensrud (24)	Quantitative descriptive	Yes	Can't tell	Yes	Can't tell	No	40%
Kristiansen and Stensrud (32)	Quantitative descriptive	Yes	Can't tell	Yes	Can't tell	No	40%
Nyberg et al. (44)	Quantitative descriptive	Yes	Yes	No	Yes	Yes	80%
Peterson et al. (33)	Quantitative descriptive	Yes	Can't tell	No	Can't tell	Yes	40%
Roth et al. (25)	Quantitative descriptive	Yes	Can't tell	Yes	Can't tell	Yes	60%
Roth et al. (34)	Quantitative descriptive	Yes	Can't tell	Yes	Can't tell	Yes	60%
Schmid et al. (35)	Quantitative descriptive	Yes	Can't tell	Yes	No	Yes	60%
Skrubbeltrang et al. (43)	Quantitative descriptive	Yes	Can't tell	Yes	Can't tell	Yes	60%
Stambulava et al. (15)	Quantitative descriptive	Yes	Can't tell	Yes	Can't tell	Yes	60%
Stornæs et al. (37)	Quantitative non-randomised	Yes	Yes	Yes	Yes	Yes	100%
Thompson et al. (38)	Quantitative descriptive	Yes	Can't tell	Yes	Can't tell	Yes	60%
Thompson et al. (39)	Quantitative descriptive	Yes	Yes	Yes	Can't tell	Yes	80%
Van Rens et al. (40)	Quantitative descriptive	Yes	Can't tell	No	No	Yes	40%
Zhao et al. (41)	Quantitative non-randomised	Yes	Yes	No	Yes	Yes	80%

Since the present study focus on quantitative results, only the quantitative part of mixed methods studies was considered for methodological quality. The study design was adapted to the subdivisions of the MMAT categories and therefore differs from the study design in Table 1. The quality ratings varied from 20% to 100% across the five assessed items, with an average score of 63%. The study quality for fifteen quantitative descriptive studies was 56% (SD = 0.18), while for six quantitative non-randomized studies, it was 77% (SD = 0.14).

In the quantitative non-randomized studies, all participants were representative of the target population, and the intervention was administered as intended. Other criteria fulfilled in the majority of studies included complete outcome data (*n* = 5) and appropriate measurements (*n* = 5). Only two studies accounted for confounders in both the design and analysis.

For quantitative descriptive studies, the sampling strategy in every study was relevant to addressing the research question. Most studies met additional criteria such as appropriate statistical analysis to answer the research question (*n* = 12) and the use of appropriate measurements (*n* = 11). Only two studies had samples that were representative of the target population (*n* = 2). Similarly, only two studies reported a low risk of nonresponse bias (*n* = 2).

No studies were omitted due to their methodological quality.

### 4.4 Outcomes on the talent-specific key aspects of LTAD

The results are presented following the talent-specific key aspects of LTAD outlined in section 2.

#### 4.4.1 Physiological

Three studies (24, 32, 41) examined physiological outcomes, including cardiovascular and metabolic parameters.

One study investigated physiological changes over a two-year period in male swimmers and racket sports athletes aged 12–14 years (41). The results of hemoglobin levels and vital capacity increased progressively, indicating a sports specific adaptation to training. Additionally, the increase was likely influenced by testosterone production, which typically rises during the onset of puberty. However, no variations were observed regarding the resting heart rate of the athletes (41).

Two studies measured the vital capacity via VO_2_max of female handball players (24, 32). In both studies the same cohort was investigated on different time points. After one year, VO_2_max results showed individual differences, but they were not significantly different between the injured and non-injured handball players. Among the non-injured athletes, the VO_2_max increased on average, while it decreased among the injured athletes (24). However, after six years all players reduced their VO_2_max ranging from 3.4% to 24.7%. The players who had no injuries or only one injury after six years experienced the least reduction in VO_2_max (32).

Overall, physiological outcomes demonstrate short-term adaptations, while long-term changes are influenced by injury status and maturation, reflecting variability across studies.

#### 4.4.2 Physical

Seven studies (25, 30, 34, 38, 39, 41, 42) examined physical outcomes by different physical performance tests. Two of the studies (30, 42) included a controlled design by comparing a second group of participants. In the sports classes (gymnastics), increased physical performance was demonstrated over the intervention period of two years compared to a regular class without sport profile (except for hand strength). Physical performance included agility, strength, flexibility, and coordination as measured by standardized fitness tests (42). Another study showed an improvement after a one-year intervention in physical performance (e.g., 20-meter sprint test, single-leg stance) of ESC students following participation in regular physical education and additional sport-specific training, compared to RS without sport profile students (30).

One study examined the development of physical performance across grade levels in students identified as having exceptional physical performance talent, using a repeated cross-sectional design. Both genders showed an increase in performance from grade 4 to grade 7, with a slightly higher rate of change observed in girls than in boys (25). In addition, a further study showed that the dynamic back strength increased over a two-year period (41). Finally, both studies from the UK demonstrated enhanced strength, speed, and power among student-athletes from a sports school (38, 39).

Only one study found a slight decrease in physical performance from boys in relation to the regular pupils from grade 4–7 whereas the physical performance of girls remained stable. The study demonstrated that the development of physical performance is influenced by sports discipline, training volume and the interaction between training volume and time (34).

Regarding anthropometric characteristics, a significant association was observed between attending sports schools and body composition (30, 34, 41). In one study at baseline, ESC students showed superior physical characteristics, including lower BMI, reduced body fat, and higher skeletal muscle mass compared to RC without sport profile students. The differences in height and weight remained after the 52-week intervention, although the differences in body fat and muscle mass were reduced (30). Longitudinal data showed age-appropriate growth patterns youth athletes, with increases in height and weight over three years (34). A further study added that Swimmers exhibited greater height, weight, and upper body development compared to racket sport athletes, though overall growth trends were similar across groups over the 96-week training period (41).

In summary, physical outcomes show short-term improvements across disciplines, while long-term trends are moderated by sport type, training load, sex, and individual differences.

#### 4.4.3 Psychological

Five studies focused on psychological characteristics (26, 27, 37, 39, 40). The results of the study by Brand et al. (27) showed that female students at an elite sports school have significantly more psychological symptoms than male students, particularly somatoform, anxiety, affective and eating disorder symptoms. Additionally, female athletes reported fewer instances of panic, post-traumatic stress, and specific phobias compared to non-athletes, though somatization was more prevalent among them (27). In contrast, deselected athletes, both male and female, showed significantly higher psychological symptoms across several categories when compared to elite athletes and non-athletes. Male athletes, in particular, exhibited higher levels of alcohol and nicotine abuse, as well as somatization and hypochondria (27).

These findings are consistent with the study by Thompson (2024), which found that female athletes have lower self-confidence in sport, more stress and greater body image concerns compared to male athletes (39). Furthermore, body image concerns were a significant issue for 17% of both genders, with some disordered eating behaviors, highlighting a potentially negative impact on their psychological health (39). In the study of Stornæs et al. (37) mental health profiles of students, categorized into “Mentally Healthy”, “Moderate,” and “Dissatisfied,” revealed that sport school boys were most often in the healthiest profile, while regular investigated girls showed the least healthy mental health profiles. Unhealthier profiles were particularly linked to socially prescribed perfectionism. Sport school students generally exhibited better mental health compared to their regular peers (37). With regard to stress symptoms in sports students at an elite sports school, no particularly high stress level was measured in relation to sports and life stress (28). The study by Earle and colleagues (26) underscores the pivotal role of Emotional Awareness in the psychological development of student-athletes. Emotional Awareness emerged as the most underdeveloped dual career competency, particularly in areas such as stress management, emotional regulation, and maintaining attentional focus. Despite being considered highly important, Emotional Awareness showed the greatest gap between perceived importance and actual ability, highlighting a critical need for targeted support in this domain to enhance long-term psychological resilience and dual career success (26).

Lastly, although talented athletes attending specialized sports schools showed lower academic motivation compared to their peers at RS without sport profile, there was no significant correlation between sport commitment and reduced school motivation. This finding challenges the assumption that high levels of sport commitment necessarily hinder academic success (40).

Taken together, the studies show that psychological risks, such as body image stress, and mental health problems, are present among student-athletes. Patterns vary by gender and school type. Female students often report higher symptom prevalence, while Emotional Awareness emerges as a consistently underdeveloped dual-career competency.

#### 4.4.4 Identity and social

Factors defining the identity, social network and surrounding of athletes were investigated by four studies (26, 35, 36, 38).

Active cross-country skiers in elite programmes had stronger relationships with coaches, higher performance and a stronger mastery orientation than those in RS without sport profile, but no differences in performance orientation and intrinsic motivation to droped out counterparts (35). Peer relationships and social support were notably strong among student-athletes, contributing positively to their overall sense of well-being (38). They felt a strong sense of belonging and acceptance within their teams. However, the balance between academic and athletic demands often left little time for socializing outside of sports, leading some athletes to lose touch with friends and family. Despite this, students reported having positive relationships with their families and a sense of autonomy in their personal lives, although their heavy schedules limited time for extracurricular activities and social interactions (38).

Furthermore, social support systems, especially from family and coaches, played a mixed role. Initially, higher levels of support were associated with better adaptation to dual-career demands, but as the year progressed, excessive support seemed to negatively affect adaptation, which could suggest the growing need for autonomy (36).

Building on the nuanced role of social support, this further emphasizes that while many athletes possess basic social competences, there remains a critical need to develop specific skills, such as collaboration with support staff and conflict resolution, that are essential for navigating the complexities of dual careers. Importantly, both studies converge on the idea that fostering athlete's autonomy alongside external support is vital, striking this balance not only enhances well-being and performance but also supports healthy identity development and sustained motivation throughout their talent development journey (26, 36).

Across studies, social support and autonomy were examined in relation to athlete development, with most studies reporting positive associations with well-being, and some indicating that high levels of support may coincide with reduced autonomy and limited social interactions outside of sport.

#### 4.4.5 Sporting success

Five studies (29, 31, 33, 40, 44) examined retrospectively the sporting success of elite athletes. It was shown that former ESC students did not achieve better athletic success at the 2004 Summer Olympics compared to their peers from RS without sport profile (29). However, a difference in athletic success between former ESC students and former regular students was documented among winter athletes (29). This may be attributed to structural differences between summer and winter Olympic sports, such as the fewer number of participating nations and the geographic and infrastructural conditions required for winter sports (29).

Another study found that athletes from ESC were more likely to win medals at international championships in the youth categories, but at the senior level, no significant differences in success between ESC graduates and those from RS without sport profile were observed (31). These results suggest that ESC did not lead to a significantly higher level of success in elite sports. Two studies showed that participation in dual career support programmes in schools (44) or attending an ESC (40) did not lead to significant differences in sporting success. In both studies, it was found that talented athletes did not achieve higher performance levels than those who attended RS without sport profile.

Additionally, a longitudinal study based on a statistical analysis was conducted to examine the differences in talent development between two cohorts of athletes at one sport school (33). The study found that the development of the groups over three, six, and hypothetically twelve years showed a gradual polarization. Initially, all students were classified as elite, but after three years, a large middle group emerged, and after six years, a large sub-elite group formed, while the elite group remained consistent but reduced (33).

Overall, sporting success outcomes show that early advantages were sometimes observed, while long-term elite performance varied across contexts and cohorts.

#### 4.4.6 Academic success

Four retrospective studies reported similar trends in academic success (30, 31, 38, 40). One study observed that athletes from ESC tended to achieve lower educational and career outcomes compared to their peers from RS without sport profile. Graduates from ESC were underrepresented in higher education and more likely to pursue vocational training or no post-secondary education. Additionally, these athletes experienced “downward educational mobility” compared to their parents and expressed lower satisfaction with their educational attainment (31). Additional, another study showed that talented athletes from RS without sport profile were more likely to achieve higher educational qualifications and university enrollment, whereas those attending ESC were significantly more likely to graduate with lower-level qualifications (40).

In contrast, no significant differences in academic success were reported between students at ESC and RS without sport profile at the beginning of the study or after one year, despite ESC athletes undergoing intense training (30). Academic grades and cognitive test scores remained consistent, indicating that ESC programmes successfully balanced sports training and academic demands (30). Although no significant between-group differences were observed in cognitive and academic performance post-intervention, the ESC group showed higher scores in all cognitive and academic measures (30). Similarly, it was highlighted that most students-athletes at another ESC maintained good academic grades despite missing classes due to training (38).

While academic success is not a core element of the LTAD approach, it remains relevant in school-based talent development and is thus included in the evaluation of sports programmes as part of a holistic perspective. In summary, ESC successfully promote physical performance without negatively affecting short-term academic success. However, long-term academic outcomes appear less favorable for ESC students compared to their peers from RS without sport profile. Complementing these findings, Earle and colleagues (26) demonstrated that athletes actively engaged in Dual Career Management and Career Planning frameworks achieved superior academic and vocational outcomes. Structured support mechanisms enabled athletes to effectively balance rigorous training schedules with academic responsibilities, thereby facilitating smoother transitions into post-athletic careers.

All in all, academic outcomes generally show short-term stability, whereas long-term achievement appears to vary according to programme structure.

#### 4.4.7 Summary across outcomes on the talent-specific key aspects of LTAD

Taken together, the studies show consistent short-term improvements in physiological and physical outcomes, whereas long-term trends display more variability. Psychological, social, sporting, and academic aspects exhibit both convergent and divergent patterns across studies. Observed variability corresponds to differences in sport type, age groups, training volume, and study design. Overall, the findings summarize trends and patterns across studies.

## 5 Discussion

This review provides a comprehensive analysis of various approaches to talent development in schools on LTAD. The analysis of 21 studies highlights the diversity of contexts and methodologies, offering valuable insights into the outcomes and limitations of talent development approaches in schools in fostering elite athletes.

### 5.1 Methodological considerations and study quality

The methodological quality of the included studies varied widely, with average scores of 63%. Although the majority of studies implemented appropriate sampling strategies, statistical analysis, and outcome measures, the lack of control for confounders in many studies and the reliance on retrospective designs in several cases limit the ability to draw definitive conclusions.

The included studies represent a range of designs, including longitudinal, repeated cross-sectional, case studies, and mixed methods, though only quantitative data were used. This reflects the complexity of talent development research, where fully experimental designs are often difficult to conduct due to practical constraints.

The focus on quantitative designs was driven by the original intent to perform a meta-analysis, requiring comparable quantitative data. This does not diminish the value of qualitative approaches, which were beyond the scope of this review.

Future research should aim to improve methodological rigor by incorporating methodologically robust cross-sectional and longitudinal cohort studies, as well as controlled studies, with particular attention to randomization, controlling potential biases, and including diverse talent development approaches across various sports and cultural contexts.

Building upon the methodological considerations outlined above, the subsequent sections further examine how talent development in school-based settings manifests across the talent-specific key aspects.

### 5.2 Talent development in schools: a Complex process

#### 5.2.1 Physiological and physical aspects: positive outcomes

The results demonstrate that talent development approaches in schools have a positive influence on physiological and physical development. Studies examining physical performance in youth athletes show clear improvements in certain areas, such as strength, speed, and endurance (30, 38). Only one study found a slight decrease in physical performance from boys in relation to pupils from RS without sport profile from grade 4–7 whereas the physical performance of girls remained stable (Roth et al. (34),). One potential explanation is that physical and anthropometric parameters do not follow a linear progression during adolescence. The hormonal changes associated with puberty significantly amplify the effects of training, particularly in male (34).

However, the variations in the effects of training, across both sports disciplines and athlete gender, point to the need for more tailored approaches in the design of talent development programmes.

Moreover, the longitudinal findings suggest that athletes attending often experience positive physical adaptations, including improved body composition and growth patterns (30, 41). From a physiological perspective, several studies revealed positive changes, such as increases in VO_2_max and vital capacity, which suggest that these athletes benefit from sport-specific adaptations to their training (24, 41).

Nevertheless, it must be noted that none of the studies reporting physiological outcomes used a controlled design. Therefore, these improvements cannot be clearly attributed to the school-based programmes alone, as age-related growth, maturation, and puberty likely played a substantial role. Age-related processes such as growth and puberty are known to significantly influence physiological performance markers such as VO₂max and vital capacity (45–47). In contrast, only two studies on physical development (30, 42) included a control group, allowing for a more robust comparison between programme and non-programme participants. These studies strengthen the assumption that talent development schools can positively impact physical performance, although more controlled research is needed to confirm these effects across physiological aspects.

Even so, the decline in VO_2_max observed in injured athletes and over a longer period highlights the importance of injury prevention and recovery strategies in maintaining LTAD (32).

#### 5.2.2 Psychological and social aspects: a diverse picture

The psychological impacts of attending talent development approaches in schools are diverse. On one hand, some studies indicate that athletes in sport schools, particularly female students, exhibit higher levels of stress and psychological symptoms, such as somatization and anxiety (27, 39). Especially female athletes were found to report lower self-confidence, greater body image concerns, and more stress than their male counterparts, raising concerns about the mental well-being of young athletes in highly competitive environments. These findings align with current research demonstrating elevated levels of anxiety, depression, somatic symptoms, and body image concerns among elite female athletes relative to their male counterparts (48), as well as among female student-athletes more broadly (48).

On the other hand, athletes at sport schools often reported better social networks, stronger peer relationships, and a sense of belonging, which have been shown to contribute positively to their mental health and well-being (35, 38). These findings highlight the importance of social support systems, particularly from family and coaches, in helping manage the dual-career pressures of student-athletes. Still, the potential negative impact of excessive support on athlete's adaptation to these demands suggests that fostering autonomy should also be a priority in talent development programmes (36).

The findings of Earle and colleagues (26) complement this nuanced picture by emphasizing the critical role of emotional awareness and social competence in buffering psychological stress and enhancing mental resilience. Specifically, athletes with higher emotional awareness were shown to better regulate negative emotions and stress responses, while strong social competence facilitated the maintenance of supportive interpersonal relationships, which are crucial for psychological well-being. These results underline the necessity of integrating psychological skills training and social development within talent programmes to promote holistic athlete health.

It should be noted that only one of the psychosocial studies included a control group. Therefore, the observed effects cannot be solely attributed to school-based talent development programmes, as other factors such as maturation and individual differences may have influenced the results.

#### 5.2.3 Sporting success: limited evidence of long-term advantages

Despite the significant investment in sport schools, the evidence regarding their contribution to long-term sporting success is inconclusive. Several studies found that school-based talent development approaches did not consistently outperform their peers from RS without sport profile in terms of Olympic or senior-level competition success (29, 31, 44). While sport schools graduates may achieve early success in youth competitions (31), there is little evidence to suggest that the benefits of specialized sports schooling translate into sustained elite performance over time [Peterson et al. (33),]. This finding underscores the complex nature of talent development, where a multitude of factors, including individual athlete characteristics, injury history, and coaching quality, play significant roles in determining long-term success.

The longitudinal analysis of athletes from one sport school indicated that early talent identification and prediction models are often inaccurate, with many athletes displaying diverse developmental trajectories over time (Peterson et al. (33),). The findings suggest either that initial predictions were incorrect in seven out of ten cases or that factors such as injuries, personal development, coaching quality, and social influences significantly impacted their progression. This suggests that talent development should not solely rely on early selection and specialized training but rather account for the dynamic and evolving nature of athlete development.

Additionally, structural factors such as early specialization, injury-related interruptions, and dropout risk were not consistently examined across studies, and most of the evidence was collected retrospectively, which limits the interpretation of long-term sporting success. Athletes who specialize early in a single sport may face higher injury rates or burnout, potentially limiting their progression to elite senior levels. Similarly, dropout or temporary disengagement from sport, often unaccounted for in retrospective studies, may distort observed success rates and contribute to the heterogeneous development patterns reported. Incorporating these structural factors in future research would allow a more nuanced understanding of the mechanisms underlying long-term sporting success.

#### 5.2.4 Academic success and career pathways: a Fragile balance

One of the more concerning findings of this review relates to the academic outcomes of students- athlete. While two studies suggest that sport schools effectively balance academic and athletic demands without adversely affecting short-term academic success (30, 38), other studies indicate that sport school students experience lower educational achievements compared to their peers in RS without sport profile (31, 40). ESC students were found to be underrepresented in higher education, more likely to pursue vocational training, and often expressed lower satisfaction with their educational outcomes (31, 40). These findings raise important questions about the long-term viability of talent development approaches in schools that prioritize sport over education and suggest that school-based talent development approaches may need to reevaluate their support structures for balancing the demands of both academic and sporting success. Both studies employed retrospective design, which limits the interpretation of their findings regarding long-term academic success.

In this regard, the study by Earle and colleagues (26) provides valuable insight by demonstrating that dual career management and structured career planning can significantly improve both academic and vocational outcomes for student-athletes. By implementing integrated support systems that allow athletes to effectively coordinate their training and education commitments, schools can facilitate more sustainable career pathways. This highlights the importance of adopting holistic, dual-career oriented frameworks within talent development programmes to safeguard academic success alongside athletic progression, addressing the fragile balance identified in this review (26).

Besides that, practical measures such as flexible curricula, academic tutoring, and individualized learning plans may help student-athletes better balance sport and education (49).

#### 5.2.5 Holistic integration of talent-specific aspects

Taken together, the findings suggest that the various aspects of talent development in schools may interact in complex ways. While the original studies did not directly examine these interdependencies, observed patterns allow for a tentative synthesis. Improvements in physical performance, such as strength, speed, and endurance, could potentially enhance athletes self-confidence and perceived competence, which may in turn support social integration and the development of supportive peer relationships (30, 35, 38). Conversely, elevated stress levels or body image concerns, particularly among female athletes, could negatively affect both social interactions and academic engagement (27, 39). Enhanced emotional awareness and social competence may buffer these risks, helping athletes to regulate stress, maintain supportive relationships, and sustain motivation in both sporting and academic aspects (26). Overall, these observations suggest that success in one aspect may reinforce outcomes in others, while challenges in one area could propagate across aspects, highlighting the potential importance of holistic and coordinated approaches in school-based talent development programmes.

### 5.3 Limitations

The primary limitation is the heterogeneity of the available evidence. This variability arises not only from the differing study designs but also from the diverse methodologies employed to assess sports talent. No study included all talent-specific key aspects, thereby complicating the comparison of findings across studies. Furthermore, the studies examined various approaches to talent development in schools and included different sports disciplines, making it challenging to draw generalizable conclusions. The training volumes were not consistently reported, and the dosage of training varied significantly across studies, ranging from 4.5 h (physical education only) (30) to 20.8 h (total training load) (41) per week. As a consequence, a meta-analysis could not be conducted, and the narrative synthesis does not provide quantitative effect estimates. Therefore, the findings should be interpreted with caution.

Many of the studies lacked control groups, and the duration of observation in longitudinal studies of student-athletes was often short, limiting the ability to assess long-term outcomes. Small sample sizes in several studies (24, 30, 32, 41) may also affect the reliability of the findings.

Another key limitation is that most of the studies included in this review focused on talent development approaches in European countries. This introduces potential cultural, social, and policy-related factors that could limit the generalizability of the findings to other countries or contexts.

Moreover, many studies did not consider additional factors such as training outside the school environment, including in clubs or private settings, which may lead to biased results. These factors could have influenced the development of the students-athletes but were not accounted for in the studies reviewed.

In conclusion, while the findings from this systematic review offer valuable insights, the limitations outlined above suggest that more research with larger sample sizes, consistent methodological approaches, and long-term follow-up is needed to better understand the effectiveness of talent development approaches in schools.

## 6 Conclusion

The findings of this systematic review provide a comprehensive overview of school-based talent development approaches and their potential impact on LTAD.

Overall, these programmes appear to positively influence athlete's physical development, social networks, and short-term academic success. However, results are mixed regarding psychological well-being, long-term sporting and academic success, with some athletes experiencing elevated stress and lower educational achievements. Additionally, talent development programmes in schools did not consistently lead to better senior-level competition success, raising questions about their effectiveness in supporting long-term athletic progression.

Overall, this review highlights the complexity of talent development in the context of schools and the need to better align talent development approaches with the aspects of the LTAD framework, especially regarding the psychosocial support as well as balance between athletic and academic development. Further research is needed to capture the long-term outcomes of these approaches on athletes and enhancing long-term success both in sport and life.

## Data Availability

Publicly available datasets were analyzed in this study. This data can be found here: No new data were generated for this study. All data analyzed were obtained from previously published studies, which are cited in the reference list. These studies are publicly accessible via PubMed (https://pubmed.ncbi.nlm.nih.gov/), Web of Science (https://www.webofscience.com/), and SportDiscus (https://www.ebsco.com/products/research-databases/sportdiscus). No repository or accession numbers apply.
