# Supplementary material for: Long-term athlete development in schools: a systematic review and narrative synthesis
Source: Front Sports Act Living. 2025 Nov 20;7:1706060. doi: 10.3389/fspor.2025.1706060 (PMC12675429; doi:10.3389/fspor.2025.1706060)
Supplement: Supplementary file 1 [file Datasheet1.pdf]

Supplementary Material

Long-Term Athlete Development in Schools: a Systematic Review and Narrative Synthesis

Nele Söker*[1], Jasper Schamerowski[1], Astrid Zech[2], Simon Steib[3], Lina Rahlf[1]

*** Correspondence:** Nele Söker: nele.soeker@uni-flensburg.de

1 Detailed search strategy

PICOS search strategy

	sport<br>AND<br>talent* OR gifted* OR high ability OR above average OR elite OR professional
**P**opulation	pupil* OR child* OR youth OR adolescence OR young OR student OR junior
**I**ntervention	program* OR test OR intervention
**C**omparison	physical education OR school sports OR talent program*
**O**utcome	performance OR conditional skills OR motor skills OR cognition OR social demographic OR development OR identification
**S**etting	school OR education OR curriculum OR academic

Boolean terms

#1	sport
#2	talent*
#3	gifted*
#4	high ability
#5	above average
#6	elite
#7	professional
**#8**	**#1 AND #2 OR #3 OR #4 OR #5 OR #6 OR #7**
#9	pupil
#10	child
#11	youth
#12	adolescence
#13	young
#14	student
#15	junior
**#16**	**#9 OR #10 OR #11 OR #12 OR #13 OR #14 OR #15**
#17	program*
#18	test
#19	intervention
**#20**	**#17 OR #18 OR #19**
#21	physical education
#22	school sports
#23	talent program*
**#24**	**#21 OR #22 OR #23**
#25	performance
#26	conditional skills
#27	motor skills
#28	cognition
#29	social demographic
#30	development
#31	identification
**#32**	**#25 OR #26 OR #27 OR #28 OR #29 OR #30 OR #31**
#33	school
#34	education
#35	curriculum
#36	academic
**#37**	**#33 OR #34 OR #35 OR #36**
**#38**	**#8 AND #16 AND #20 AND #24 AND #32**

2 Documentation of database search

Pubmed

Date: 16.04.2025

Filter: none

Search mode: All Fields

Result: 5582

Web of Science

Date: 16.04.2025

Filter: none

Search mode: All Fields

Result: 4046

SPORTDiscus

Date: 16.04.2025

Filter: none

Search modes: Abstract or Author-Supplied Abstract

Result: 215
